# Co-Simulation of Interconnection Between Smart Power Grid and Smart Cities Platform via Massive Machine-Type Communication

**DOI:** 10.3390/s25051517

**Published:** 2025-03-01

**Authors:** Luiz H. N. Rodrigues, Carlos F. M. Almeida, Nelson Kagan, Luiz H. L. Rosa, Milana L. dos Santos

**Affiliations:** 1 Electrical Engineering Department, Universidade de São Paulo, Sao Paulo 01000-000, Brazil; cfmalmeida@usp.br (C.F.M.A.); nelson.kagan@gmail.com (N.K.); milanals@usp.br (M.L.d.S.); 2 Instituto Federal de São Paulo Electrical Engineering Department, Sao Paulo 01000-000, Brazil; luizhenrique@ifsp.edu.br

**Keywords:** co-simulation, smart power grids, smart cities, massive machine-to-machine-type communication, distributed computing

## Abstract

With the advent of Industry 5.0, the electrical sector has been endowed with intelligent devices that are propelling high penetration of distributed energy microgeneration, VPP, smart buildings, and smart plants and imposing new challenges on the sector. This new environment requires a smarter network, including transforming the simple electricity customer into a “smart customer” who values the quality of energy and its rational use. The SPG (smart power grid) is the perfect solution for meeting these needs. It is crucial to understand energy use to guarantee quality of service and meet data security requirements. The use of simulations to map the behavior of complex infrastructures is the best strategy because it overcomes the limitations of traditional analytical solutions. This article presents the ICT laboratory structure developed within the Department of Electrical Engineering of the Polytechnic School of the Universidade de São Paulo (USP). It is based on an architecture that utilizes LTE/EPC wireless technology (4G, 5G, and B5G) to enable machine-to-machine communication (mMTC) between SPG elements using edge computing (MEC) resources and those of smart city platforms. We evaluate this proposal through simulations using data from real and emulated equipment and co-simulations shared by SPG laboratories at POLI-USP. Finally, we present the preliminary results of integration of the power laboratory, network simulation (ns-3), and a smart city platform (InterSCity) for validation and testing of the architecture.

## 1. Introduction

We are currently witnessing the advent of Industry 5.0, which was estimated to enable approximately 50 billion devices to be connected via the Internet of Things (IoT) by the end of 2023 [1]. There are more than 1.8 M2M (machine-to-machine) connections for every person on the globe, which is a significant figure [2]. As a result of this revolution, the electrical power sector was also equipped with intelligent technology, which led to a significant increase in distributed and non-programmable electrical power microgeneration, smart buildings, smart homes, and smart meters (SM) [3]. This, in turn, has created new challenges for the operation of energy systems. This new environment calls for a smarter network, including the transformation of the traditional energy electricity customer into a “smart client” and, in many cases, into a “smart prosumer.” This has, in turn, led to the emergence of new challenges for the operation of power systems.

It becomes clear there is a need for a smarter grid [4]. InterSCity is a platform for smart cities that was developed by USP within a multidisciplinary project that uses digital technologies to make all city services more efficient and reliable, including the supply of electricity [5]. The implementation of the electric energy vertical on smart city platforms through applications (load prediction and monitoring, among others) in SPG requires (1) data analysis algorithms to evaluate the data generated by intelligent devices and meters and (2) performance evaluation of the various possible architectures to interconnect all SPG elements at the link and physical layer level. On the other hand, to connect these systems, a communication backbone is needed. Smart grids present many different communication challenges, and 5G cellular network technology helps address these challenges because it was designed to meet the heterogeneity of this demand [6]. Recent technologies, like MEC, NFV, and SDN, help 4G and 5G cellular networks [7,8].

**Figure 1:**
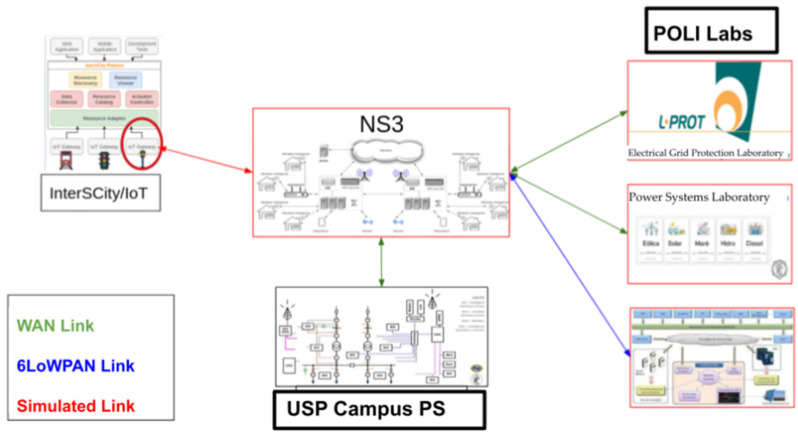
Our proposed integration architecture that uses data generated by POLI laboratories (POLI Labs: L-PROT, Lab-SISPOT, and NAPREI), the USP campus PS (power substation), and the smart cities platform (InterSCity) through ns-3 simulation, which implements mMTC via MEC for applications that manage SPG functionalities.

In this sense, an architecture is proposed (Figure 1) based on MEC (multiaccess edge computing) and LTE (long-term evolution) technologies that allow mMTC (massive machine-type communications) between SPG components, legacy software resources, and smart city platforms. Our architecture was evaluated through simulations and emulations using data from real and simulated devices (Paravirtualization, HIL, and SIL) shared by the SPG laboratories at POLI-USP (NAPREI—Research Support Center at SPG, L-SISPOT—Power Systems Laboratory, and L-PROT—Electrical Grid Protection Laboratory [9,10,11,12,13,14]), in São Paulo, Brazil. The use of data generated by simulators and emulators available in these laboratories opens a range of options for performance and interoperability tests of the various possible solution architectures in the interconnection of applications and these network elements that meet the demands of “prosumer” customers [15,16]. These options are intended to explore communication (mMTC, MEC, B5G—beyond 5G) between real equipment (IEDs—intelligent electronics devices, SMs, etc.) that make up the network of devices installed in the laboratories.

The rest of this paper is divided as follows: Section 2 describes the objectives of this proposal. Section 3 describes the methodology used in developing the work. A survey of related works is presented in Section 4, and a description summary of the systems studied, that is, SPG and the interaction between the NAPREI laboratory and the architectural blocks and LTE, mMTC, and the InterSCity platform will be covered in Section 5. Section 6 presents the results of utilizing the laboratory to test and validate the architecture’s functionalities, and Section 7 presents the conclusions and future work.

## 2. Goals

This work presents a new system architecture that guarantees reliability and latency for SPG using a co-simulation platform that exploits a paravirtualized simulation approach to evaluate the performance of the integrated SPG with legacy information systems and simulated data networks (LTE and mMTC). This approach allows access and management of the devices necessary to enable the connection of smart microgrids, smart homes, smart buildings, smart factories, etc., in the context of smart cities.

## 3. Proposed Methodology

The steps that guided the methodology in this work were as follows:

1. Definition and execution of a series of benchmarks to evaluate the best experimental environment to perform simulations and emulations of LTE network communications in an SPG environment;
2. Survey of architecture proposals previously found in the literature and evaluation of them in terms of refined monitoring of the electrical grid and services for SPG systems;
3. Adaptation of the best architecture from the previous item so that it can support massive machine-to-machine communication expected in SPG environments;
4. Modification of the previous version of the InterSCity platform to support SPG applications with QoS guarantees.

## 4. Related Works

Simulations and experiments are crucial across various application subdomains within smart grids [11]. They offer a valuable opportunity to test recent technologies, models, and methodologies in a controlled setting before their implementation in real-world scenarios. The availability of vast amounts of data collected from smart grids has enabled many research approaches. In ref. [11], Rossi et al. conducted an extensive literature survey with the aim of gaining insights into different facets of data communication in smart grids, namely, load control, forecasting, clustering, tool support, experiments/simulations, and replicability, in addition to research reproducibility. According to Gavriluta et al. [17], regarding the communication system and distributed computing used in SPG simulation studies, in most of the previous literature, the communication and distributed computing aspects are ignored or missing. Therefore, in this survey, we decided to evaluate the available bibliography separately for data networks, electrical power grid simulators, and co-simulation with both systems, as presented in the following subsections. This bibliographical survey helped to create the methodology presented in this work, the importance of data communication simulation for smart grid laboratories, and many ways to study smart grids. So, there is room for innovative ideas in testing tools, communication protocols, and new smart grid features.

### 4.1. Data Network Simulator

In our literature review, several data network simulators were studied, and after selection, the network simulator ns-3 was tested. The ns-3 is a communications network simulator widely used in academia because it represents network behavior very close to the real thing and has a very active and connected community of developers, in addition to being open source. MATLAB 2024 software (R2024a) [18], together with its Simulink module, is very present in most theoretical scientific articles in the sense of a numerical and statistical approach to the various characteristics of communication technologies, such as channel capacity, frequency response, and quality prediction services [19], but without the flexibility of real-time simulations. Co-simulation environments for SPG are often preferred in the academic literature because of their enhanced flexibility, allowing the integration of specialized tools tailored to specific aspects of a system, thereby providing a more accurate and efficient simulation experience. The pure multi-domain simulators (e.g., MATLAB 2024/Simulink, Modelica [20,21]) are solutions to a specific tool, have restricted use, and do not show much friendly integration. The OMNeT++ simulator is composed of modules, libraries, and extensible C++ simulation procedures, which can work in real-time, and the simulator is distributed under the Academic Public License; this fact does not satisfy our criteria for using open-source technology. In ref. [22], Albagli et al. used the SPG co-simulation framework using high-level architecture (HLA) together with federated MATLAB/Simulink, simulated the electrical models, and sent attributes to the interface with the runtime infrastructure, in real-time, while the federated OMNeT++ was responsible for receiving the messages from the federated JADE agents and forwarding them to the rest of the system. QualNet was developed to help in the designing and simulation of communication networks [16,23]. According to the manufacturer’s official website [24], although it is not sold, SCALABLE software needs a license agreement to be used. The OPNET network simulator [25] has a fixed set of protocols/devices, so users cannot create new protocols or modify the behavior of existing ones, that is, despite being a simulator widely used by communication system designers, OPNET does not allow the flexibility necessary for the proposal of this article.

### 4.2. Electric Power Grid Simulators

Strasser et al. [26] presented a study on the difficulties faced by researchers who carry out studies with co-simulation, and a classification was also made for the different simulation modalities, which were analyzed. In classical simulation, a single respective tool was used to integrate the model equation, representing the entire operation of the studied system. In their article [27], Duy Le et al. presented co-simulation as necessary to model the interaction between the components of so-called CPS (cyber–physical systems). In the study, a general survey of several co-simulations for SPG research was carried out. They also concluded that the integrated use of the FCNS, GridLAB-D, and ns-3 simulators was a great contribution to research on smart grids, surpassing the co-simulation speed of other proposals by up to 20% [28]. Applying these tools and the IEEE standard model composed of 13 power buses (IEEE—13 buses), a study was carried out on threats, security, demand/response, and dynamic pricing for SPG. The authors also suggested using this tool for cybersecurity training [28]. Kelley et al. [29] presented, with FSKIT, a set of federated tools that perform a co-simulation of power and communication networks. To simulate the electrical network, a dynamic simulator of electrical power systems driven by high-performance computing (HPC), GridDyn, was used. Zhang et al. [30] discussed a typical SPG cyber physical system for monitoring and control of distributed energy resources (DERs). The integration of ns-3 with the energy network simulator, HELICS (Hierarchical Engine for Large Scale Infrastructure Co-Simulation) was proposed by the authors in an open-source cyber physical energy co-simulation platform. A case study was analyzed using an abstraction that unites common codes between several software projects, providing the generic functionality, framework of co-simulation, distribution, and high-performance communication based on HELICS for the coordination of DER. The co-simulation framework for ns-3 that integrates with HELICS was developed by the authors. Souza et al. [31] used the Mosaik framework to study SPG integrated with a power flow simulator. Venkataramanan et al. [32] presented a cyber physical test environment of loads in a microgrid in a co-simulation of the power system (RTDS) and emulation of a TCP/IP. An architecture adopted by Trajano et al. [33] makes use of the LTE/EPC module from the ns-3 simulator and implements MEC for 4G and 5G mobile cellular networks. This architecture demonstrates that it meets the great demands for an SPG deployment designed by electricity sector forecasts. Although it is not a co-simulation, the ease that ns-3 has in performing paravirtualization provides excellent possibilities for creating testbeds capable of allowing relevant studies in SPG.

### 4.3. Electric Power Grid and Data Communication Co-Simulations

Table 1 outlines how our proposal compares to previous smart grid co-simulation projects. The comparison covers several important criteria and shows our main contribution of co-simulation, which is to demonstrate the possibility of integration between smart city platforms and SPG labs via LTE cellular technology through the network simulator (ns-3). The EPOCHS platform [34] was indeed a breakthrough, as it allowed for integrated simulation of these two critical infrastructures and SPG and enabled better insights into how communication networks can affect the stability and performance of power systems. Gaouda et al. [35] used the Hampden 180 to simulate a power system on a reduced scale, using various electromechanical equipment. Kim et al. [36] presented a co-simulation framework that uses OPNET, a communication network simulator, and OpenDSS Version 10.1.0.1, a power system simulator applied to study demand response applications within smart grid scenarios. In the work by Mirz et al. [37], the authors proposed a co-simulation architecture designed to focus on the integration of power system communication and smart grid market analysis. In ref. [4], Barbierato et al. proposed a distributed multi-model co-simulation platform for SPG that exploits the communication paradigms of IoT platforms. In the architecture of the NAPREI’s smart power grids laboratory [9,10], applications were developed in an innovative smart grid emulator, which is the best experimental environment to perform simulations and emulations of LTE network communications. This structure used only a 6LoWPAN for data communication, a fact that was a problem when real-time simulations were needed, as it did not represent the real situations encountered in the day-to-day operations of an energy company. The current proposal corrects this gap, a need that was highly requested by the market.

## 5. Description of Co-Simulated Systems

This section describes the implementation of A co-simulation of an SPG environment in the context of smart cities. The individual systems are described below.

### 5.1. Smart Power Grids (SPG) and NAPREI Laboratory

**Figure 2:**
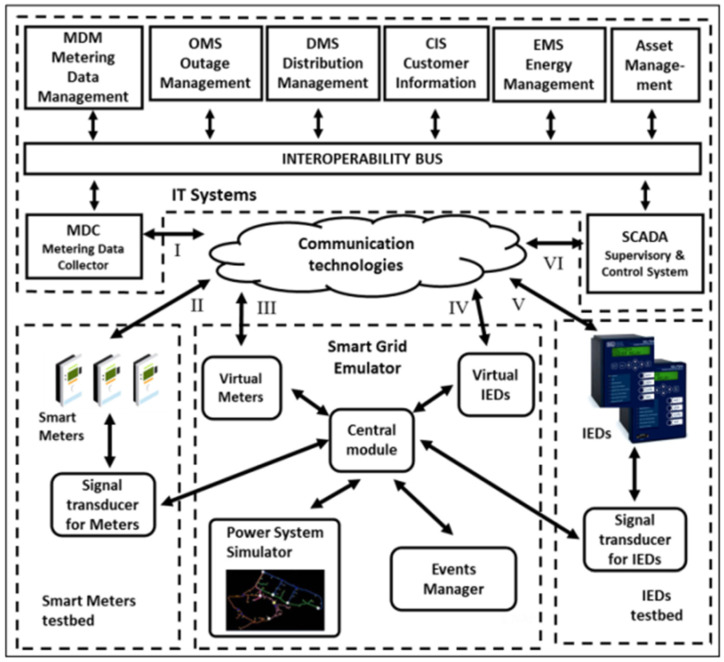
Block diagram of the NAPREI’s SPG laboratory with emphasis on elements (I, II, III, IV, and V) of each research block. Source: [9].

According to Kagan [38], SPG must integrate automation, intelligent measurement and actuation systems, and distributed energy resources. Currently, the SEP (electrical power system) needs to provide functionalities to meet specific objectives and, therefore, requires ICT (information and communication technology) infrastructure. To manage such intelligent and complex electrical networks, SPG applications use advanced control strategies [39]. JRC’s “Smart Grid Laboratories Inventory 2020” report [40] states that the infrastructure necessary for research in the sector is of vital importance in validating prototypes and their solutions. The interoperability of new computer systems introduced and the performance evaluation of SPG are also proven by this infrastructure. In this sense, NAPREI developed an SPG emulator to study and test a range of functionalities of IEDs and SMs that need to interact with legacy distribution management systems, that is, SCADA, MDM, and DMS systems, among others. In ref. [9], Rosa presents in detail the SPG emulator and states that it proposes an original and innovative form of systemic testability of SPG functionalities in a controlled environment, as it includes IEDs, a measurement island, telecommunications infrastructure, and legacy systems of information, which are integrated into a diversity of simulations in complex and non-simplified electrical energy distribution networks. The block diagram of NAPREI’s SPG laboratory is presented in Figure 2, in schematic form, covering the research objects. The REI emulator (I) was developed for systemic testing of SPG functionalities involving equipment (hardware) and computer systems (software) in the laboratory environment. The measuring island (II) consists of load/generation emulators (Emuladores de cargas e geração), smart meters (Medidores Inteligentes), and communication infrastructure, and is capable of representing any consumption/generation situation of an electrical energy consumer belonging to an electrical distribution network. The telecommunications infrastructure (III) provides the interconnection of the whole system. The use of physical intelligent electronic devices (IEDs) (IV) is a fact of great importance in REIs, as they are increasingly making use of new systems spread across distribution networks and the IT systems (V).

### 5.2. Communication Systems

As can be seen in the central part of Figure 2, the telecommunications infrastructure (III) provides all the interconnection of the emulator (I), the measuring island (II), the IEDs (IV) to the center, and the IT systems (V). In our work, this functionality is carried out by connecting via LTE, as presented in the next section. The ability to interconnect a heterogeneous network (HetNet) is one of the advantages of using 5G that uses LTE, as it allows a connection with a macro-cell (Macro BS), that is, through a radio base station (RBS ou eNB, and in Portuguese ERB—*Estação Rádio Base*), to a connection through a Femtocell. The purpose of this study is to develop a communication architecture that serves as an IoT interface for NAPREI laboratory simulations by providing an energy control system perspective on the implementation of global services through SPG systems, such as residential load prediction and NILM (non-intrusive load monitoring). Thus, this architecture is intended to be the basis for future implementation of the energy vertical for smart city platforms.

The electricity sector is one of the most challenging “test cases” for 5G cellular mobile networks, as it has a huge number of different requirements that need to be addressed, such as managing smart metering and low latency in fault locations. There are still several issues related to automation, security, resilience, scalability, and portability of 5G network management. As one can see in Figure 2, typically, the literature just describes the needs of a communication system. Rossi et al. [11] presented several challenges of smart networks in the interconnection between the physical infrastructure with ICTs (information and communication technologies). One of today’s challenges dealing with the explosion in wireless traffic is the deployment of many small cells giving rise to networks. MEC is the most frequently indicated technology to support smart city needs [41], as its multi-access allows the connectivity of a wide variety of devices, including wired interfaces and Wi-Fi (GPRS/UMTS/LTE), simultaneously. Kagan [38] presented telecommunications in different communication protocols, that is, a heterogeneous network, so the proposal of this subproject is precisely to use 4G LTE to support 5G and thus carry out all communications in all protocols. The cloud communication technologies in Figure 2 are the focus of our proposal. Data transfer latency provoked by the security system (blockchain) is a very important theme in a system that integrates several heterogeneous components, like SPG [42].

### 5.3. Smart Cities Platform—InterSCity

**Figure 3:**
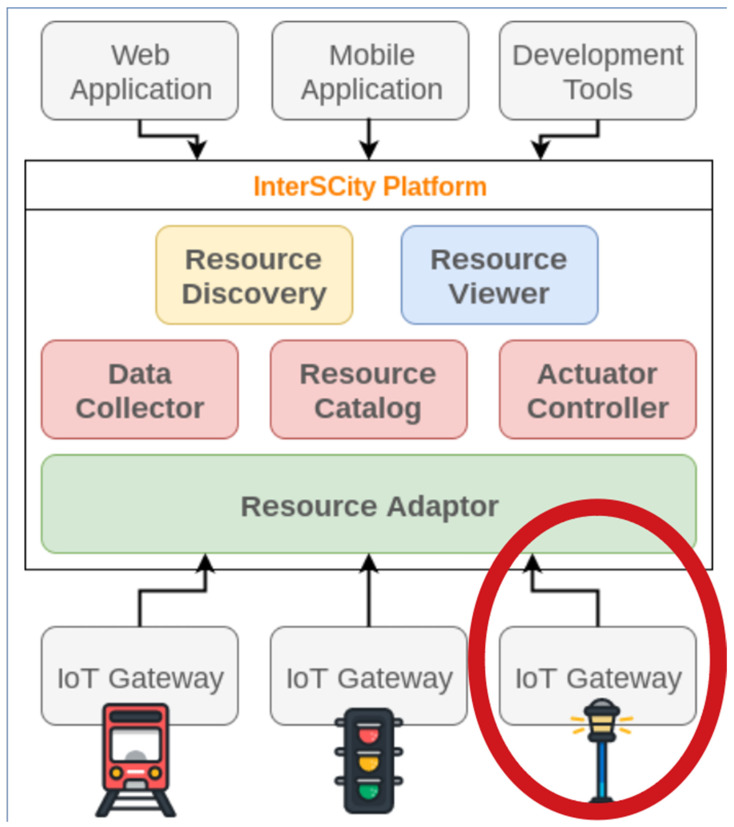
InterSCity is a web-based microservice. IoT gateway for SPG is emphasized in the red ellipse.

The JRC report [43], which discusses smart grid labs, highlights that the theme of “smart cities” and its nature of connecting different layers of technology demand complete and holistic solutions, which adds value to the smart grid sector. The report highlights the increase in investments in smart grid labs and that there is no forecast of a reduction in the number of active labs in this area. The InterSCity platform is designed to simplify the management and integration of smart city services by offering a suite of high-level, web-based micro-services, as described in Figure 3 [4]. These services afford the necessary tools to handle the IoT technologies, enabling the finding of city services and devices, storing and processing data, and intermediating action commands. The platform supports a range of smart city applications across various domains, known as verticals, with a specific example being electrical energy vertical as the REIs in Figure 2. By mediating data transfer between city applications and services for citizens, InterSCity abstracts the complexities involved in city-scale data management and the specific communication protocols required by the underlying IoT devices. This abstraction layer ensures that users can focus on developing and deploying applications without needing to worry about the intricate details of the city’s infrastructure.

## 6. Results

Rodriques and Almeida [44] presented, in Portuguese, the preliminary results of the LTE/EPC cellular simulation for integration of laboratories for validation and testing of the architecture. However, in this article, it was decided to evaluate the available systems separately for data network and electrical network simulators, as presented in the following subsections.

### 6.1. LTE/MEC Data Network Simulation

**Figure 4:**
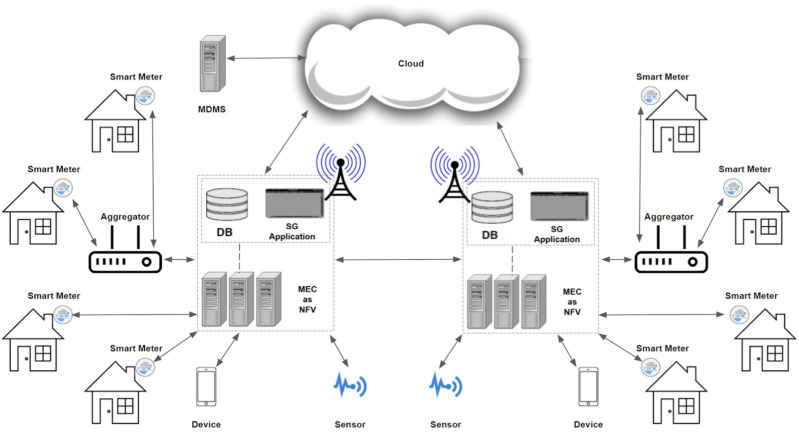
MEC applications in simulation eNB’s.

In their article [33], Trajano et al. proposed and evaluated an architecture based on MEC (mobile edge computing), which is efficient in meeting reliability and latency demands. This considers the distributed applications for SPG via mobile cellular networks utilizing LTE/EPC (4G and B5G). The authors used ns-3 to demonstrate the proposal’s ability to handle a realistic number of SMs, supporting many SPG deployment use cases. MEC is a technology standardized by the ETSI (European Telecommunications Standards Institute) [8]. In our data network simulation, there is a similar proposal, as shown in Figure 4.

**Figure 5:**
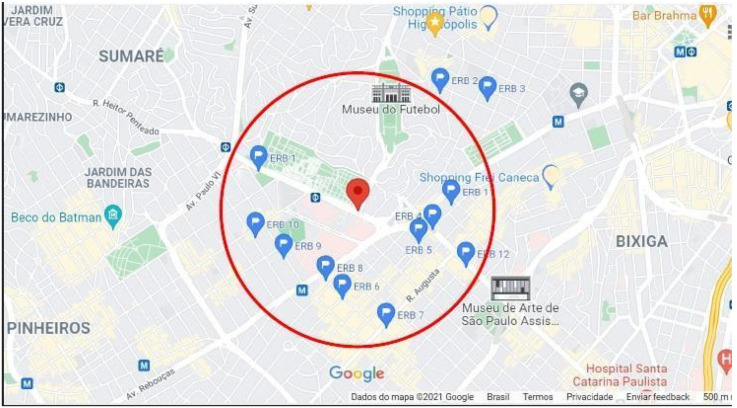
A Google Maps^®^ view of the Cerqueira César neighborhood (São Paulo—SP) utilized in the simulation. The BSs are marked by blue pin icons with white flags, while the red pin denotes the central area of interest for the experiments.

To revalidate the simulation in this article, mainly determining whether the simulated network can support many SPG devices, the LTE network was set up using the parameters listed in the ITU-R M.2135-1 report [45]. In the experiments, the topologies extracted from deployments are used with real LTE/EPC from neighborhoods in the cities of São Paulo, Brazil, which is one of the largest cities in Latin America and has 11.4 million inhabitants with 7528.26 hab/km^2^ population density, according to the last census [46]. Figure 5 shows the actual locations of the base stations (ERBs) of a cellular mobile telephone operator on a map implemented from Google Maps, in São Paulo. The Cerqueira César neighborhood used in the simulation, located between the neighborhoods of Jardins da Bandeira, Sumaré and Bixiga. This region includes a big shopping center (Shopping Pátio Higienópolis) and two famous museums: football (Football Museum) and art (MASP—São Paulo Art Museum).

**Figure 6:**
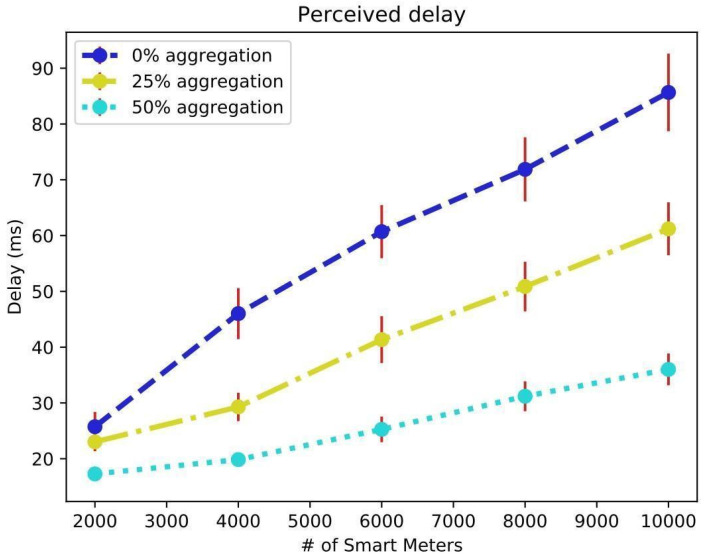
Average message delay (in milliseconds) sent from multiple smart meters to MEC server in simulation of 0%, 25%, and 50% aggregation.

The experiments were conducted using ns-3 version 3.28, leveraging its built-in LTE/EPC module, which provides robust support for LTE-based simulations. The setup involved a machine equipped with an Intel Core i5 8th Generation processor, 12 cores, and 4 GB of RAM. From the central limit theorem (95% confidence interval is used to calculate the error), the margin repeats all simulations 30 times. Each process for the different scenarios took about 5 h to complete. Smart devices and meters had their positions randomly generated within a circle with a radius of 1 km from the center of the chosen region (InCor Hospital) and represented in ns-3 with fixed positions using model-defined ConstantPositionMobilityModel. The coordinates for the LTE BS were sourced from those installed in the city (Figure 4) in alignment with the macro urban cell scenario. Figure 6 illustrates the average perceived delay, measured in milliseconds, in communication between devices and the MEC servers. The aggregation factor (AF) (percentage of SMs connected via concentrators) is a crucial parameter to consider for designing a robust intelligent network deployment, as demonstrated in Figure 6.

In Figure 6, as observed in the scenario with 10,000 SMs without any aggregation device, the delay of 90 ms for application requirements is deemed high for certain SPG use cases. According to IEC, this value is not accepted for services proposed for communication in SPG [47,48]. However, with aggregation, the delay decreased. For an AF of 25%, the delay dropped to 60 ms, and for an AF of 50%, the delay dropped to 35 ms, which are considered acceptable [49,50].

**Figure 7:**
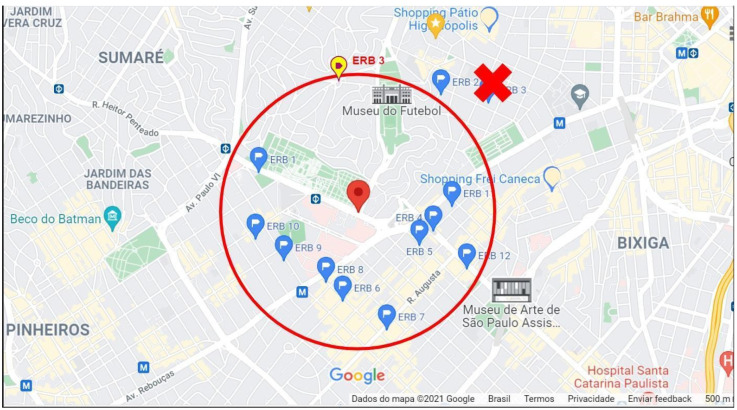
Relocation of ERB 3 within the region of interest.

As can be seen in Figure 5, there is a relatively large region in the upper part of the circle surrounding our area of interest, including the center of the circle and the Football Museum, which is serviced by eNBs very far apart (ERB 1 to 5 and 11). Therefore, after a simple cellular service optimization technique, a relocation was proposed for the eNB identified as ERB 3. In Figure 7, the ERB 3 actual position is marked with the red “X”, and the new proposal position is denoted with the yellow pin inside the red circle.

**Figure 8:**
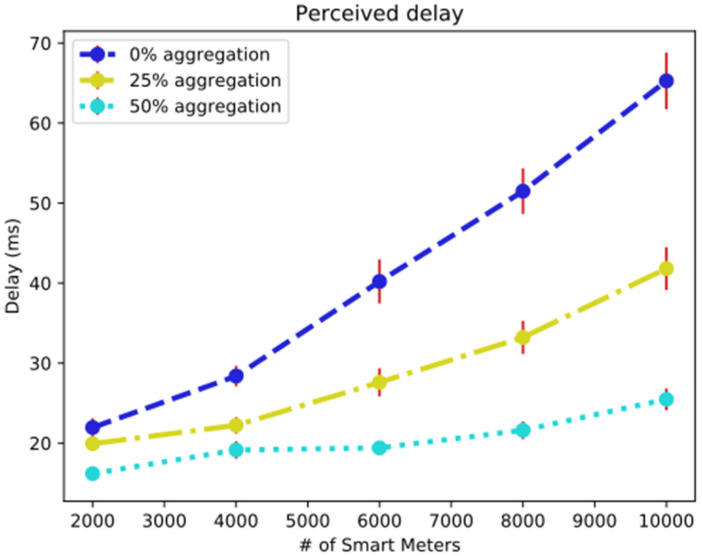
Average message delay after the relocation of ERB 3.

Figure 8 shows how effective the relocation was; the average delay perceived in milliseconds reduced to about 65 ms without aggregation. For an AF of 25%, the delay dropped to 43 ms, and for an AF of 50%, the delay dropped to 25 ms. These values are acceptable in the services proposed for SPG.

### 6.2. Integration of the InterSCity Platform Between SPG and Other Smart City Verticals

In ref. [5], del Esporte et al. focused on the architecture of InterSCity, highlighting its flexibility, extensibility, and scalability. They presented experimental results that demonstrate how well the platform can scale, likely in various urban or IoT-based scenarios.

In ref. [51], Viana et al. provided an experience report on the software engineering practices involved in developing applications using the InterSCity platform. This likely includes insights into the development of the lifecycle, challenges faced, and how these practices impacted the creation of smart city solutions. In our experiments, we show how the platform can serve several competing verticals without presenting any scalability problems. During three periods (2023–2024), 17 computer engineering undergrad students’ teams used InterSCity and developed their projects, creating software documentation in GitHub repositories [52,53,54,55,56,57,58,59,60,61,62,63,64,65]. Those projects were deployed in only one instance of the platform, at the Intelligent Distributed Systems Laboratory (LSDi—Laboratório de Sistemas Distribuídos Inteligentes) of the Universidade Federal do Maranhão (UFMA). Several projects of different verticals of service provision for smart cities were implemented. As a study of NILM, the Hedwig project [66], which is developed by students from the POLI-USP postgraduate program, integrates grouped data with the InterSCity API [67,68]. After collecting real data from September 22nd to October 21st, 2020, in a connected home testbed in the city of Santo André (10 PIR motion sensors, 10 lamp status sensors, and 8 non-current sensors, invasive—total consumption—and 4 sectors—stored in modules installed in the connected home), pre-processing (one-hot encoding) and data grouping were carried out with unsupervised learning algorithms. The k-means algorithm performs the grouping of movement events and lighting status of the connected home.

In terms of scalability [5] and security [42], other experiments with the InterSCity platform using a blockchain-based management model were conducted. These experiments have proven that the InterSCity platform perfectly meets the requirements demanded for SPG.

## 7. Conclusions and Future Works

This work presents massive machine-to-machine communication applications for SPG on a smart cities platform (InterSCity), which uses data generated by the POLI-USP Laboratories (NAPREI, L-SISPOT, and L-PROT) to implement mMTC architecture via MEC for the InterSCity platform as applications that manage SPG functionalities. The most significant contribution of this work is filling the gap found in network integration of electrical and telecommunications infrastructure necessary for its management and operation. The use of co-simulation demonstrates the effectiveness of the proposed solution without the need to physically implement any equipment in the field, which would be expensive and traumatic in the operation of a real electrical network. The use of NAPREI simulators and emulators opened a range of options for performance and interoperability testing of the various possible solution architectures in the interconnection of applications and smart microgrids, smart buildings, smart electronic devices (IDEs), and smart meters. Of these possibilities, we highlight the generation of data for the future development of testing applications in big data to access the levels of real off-shore equipment (IDEs, smart meters, etc.), emulations/simulations of specific configurations for scalability testing, heterogeneity, data management, privacy, and security, among other challenges that are intended to be implemented in a vertical of smart electric grids on the InterSCity platform, including the implementation of global energy management. It is proposed, as one of the most important future works of the proposal, to replicate this prediction methodology in the NAPREI network. The project’s proximity to NAPREI is a very promising partnership to add to the expertise of SPG laboratories and the InterSCity platform. The various advances achieved at NAPREI demonstrate the laboratory’s capacity and that the development of global management is one of the possibilities envisioned in a partnership. Paravirtualization is the hybrid of co-simulation and virtualization. This technology is very interesting because it has all the elements developed so far in research and can then be integrated into the physical infrastructure of the NAPREI laboratory, that is, all its equipment and ICT equipment (NAPREI software) and the data network simulator paravirtualization modules simulated in ns-3. Integration of the physical infrastructure of the NAPREI laboratory with two other laboratories at POLI-USP, namely, L-PROT (Protection Laboratory), which includes a real-time digital simulator (RTDS), and the Power Systems Laboratory, which includes four mini generation systems with digital interface, was implemented with NI’s LabView2024 software [69].

## Figures and Tables

**Table 1:** Comparison between our co-simulation infrastructure and the literature’s solutions.

Reference	Simulator	Net Simul	HIL	IoT	Smart City
[34]	PSCAD/EMTDC PSLF	ns-2	No	No	No
[35]	Hampden 180	LAN	No	No	No
[36]	OpenDSS	OPMET	No	No	No
[37]	Python Modelica	ns-3	No	No	No
[4]	Python MATLAB Simulink	ns-3 Mininet Omnet++	Yes	Yes	No
[9,10]	NAPREI	6LoWPAN	Yes	No	No
Our Proposal	NAPREI InterSCity	ns-3	Yes	Yes	Yes

## Data Availability

The original contributions presented in this study are included in this article; further inquiries can be directed to the corresponding author.
